# Supplementary material for: Paracrine Met signaling triggers epithelial–mesenchymal transition in mammary luminal progenitors, affecting their fate
Source: eLife. 2015 Jul 13;4:e06104. doi: 10.7554/eLife.06104 (PMC4498445; doi:10.7554/eLife.06104)
Supplement: Supplementary file 1. — Regenerative capacity of cells isolated from untreated and HGF-treated primary spheres. DOI: http://dx.doi.org/10.7554/eLife.06104.026 [file elife06104s007.docx]

| Transplanted cells | Number of cells transplanted | | |
| --- | --- | --- | --- |
|  | 2000 | 1000 | 500 |
| Untreated Lu-pos | 1/2 | 0/2 | 1/4 |
| HGF-treated Lu-pos | 2/2 | 1/2 | 4/4 |

Supplementary File 1: Regenerative properties of HGF-treated and untreated cultures of luminal progenitors identified by ICAM-1. Results are expressed as the number of successful outgrowths out of the number of transplanted fat pads.
