# Supplementary material for: Paracrine Met signaling triggers epithelial–mesenchymal transition in mammary luminal progenitors, affecting their fate
Source: eLife. 2015 Jul 13;4:e06104. doi: 10.7554/eLife.06104 (PMC4498445; doi:10.7554/eLife.06104)
Supplement: Supplementary file 2. — List of primers for q-PCR. DOI: http://dx.doi.org/10.7554/eLife.06104.027 [file elife06104s008.doc]

# Supplementary file 2: List of primers for q-PCR

Cdh1-s 5’- ATCCTCGCCCTGCTGATTC - 3’

Cdh1-as 5’- ACCACCGTTCTCCTCCGTA - 3’

Csn2-s 5’- CCTCTGAGACTGATAGTATTT - 3’

Csn2-as 5’- TGGATGCTGGAGTGAACTTTA - 3’

Elf5-s 5’- CCAACGCATCCTTCTGTGAC - 3’

Elf5-as 5’- AGGCAGGGTAGTAGTCTTCA - 3’

Esr1-s 5’- CTGGACAGGAATCAAGGTAAA - 3’

Esr1-as 5’- GAGGCACACAAACTCTTCTC - 3’

Gapdh-s 5’- CCAATGTGTCCGTCGTGGATC - 3’

Gapdh -as 5’- GTTGAAGTCGCAGGAGACAAC - 3’

Gata3-s 5’- AGGCAGGGAGTGTGTGAAC - 3’

Gata3-as 5’- TTCGCTTGGGCTTGATAAGG - 3’

Icam1-s 5’- GACCCCAAGGAGATCACATT - 3’

Icam1-as 5’- CCTCGGAGACATTAGAGAAC - 3’

Krt18-s 5’- CCTTGCCGCCGATGACTTTA - 3’

Krt18-as 5’- CAGCCTTGTGATGTTGGTGT - 3’

Krt5-s 5’- GACCAGTCAACATCTCTGTC - 3’

Krt5-as 5’- TGCCAACACCAATGCTGCTG - 3’

Krt6a-s 5’- GAAGCAGAACTGTCTCAGATG - 3’

Krt6a-as 5’- GTCCAGGCTACGGTTGTTG - 3’

Ly6a-s 5’- GTCCCATTTGAGACTTCTGT - 3’

Ly6a-as 5’- TACCCAGGATCTCCATACTT - 3’

Mki67-s 5’- TCAGATGGCTCAAAGAACAGT - 3’

Mki67-as 5’- CAGAAGTGGGCTCTACTATG - 3’

Pgr-s 5’- CCACCTGTACTGCTTGAATAC - 3’

Pgr-as 5’- CAACTGGGCAGCAATAACTTC - 3’

Prlr-s 5’- ATAAAAGGATTTGATACTCATCTG - 3’

Prlr-as 5’- GTCATCCACTTCCAAGAACTC - 3’

Tnfrsf11a-s 5’- TTGGACACCTGGAATGAAGAA - 3’

Tnfrsf11a -as 5’- CACACCGTATCCTTGTTGAG - 3’

Tnfsf11-s 5’- CAAGATGGCTTCTATTACCTG - 3’

Tnfsf11-as 5’- ATGCTGGTTTTAACGACATAC - 3’

Snai1-s 5’- ACACCTGTTTCACAGCAGTT - 3’

Snai1-as 5’- TAGTTCTGGGAGACACATTG - 3’

Snai2-s 5’- GATGCCCAGTCTAGGAAATC - 3’

Snai2-as 5’- CCCAGTGTGAGTTCTAATGT - 3’

Trp63-s 5’- TGCCCAGACTCAATTTAGTGA - 3’

Trp63-as 5’- GAGGAGCCGTTCTGAATCTG - 3’
